# P-682. The Risk of Cardiorespiratory Events for up to 180 days Following Respiratory Syncytial Virus (RSV) Infection Hospitalization: A Self-controlled Case Series Analysis

**DOI:** 10.1093/ofid/ofae631.878

**Published:** 2025-01-29

**Authors:** Caihua Liang, Jennifer Judy, Negar Aliabadi, Reiko Sato, Erica Chilson, Yun Zhou, Xin Zhao, Bradford D Gessner, Elizabeth Begier

**Affiliations:** Pfizer Inc, New York, New York; Pfizer, New York, New York; Pfizer, New York, New York; Pfizer, Inc., Collegeville, Pennsylvania; Pfizer, New York, New York; Genesis Research Group, Hoboken, New Jersey; Genesis Research Group, Hoboken, New Jersey; Pfizer Biopharma Group, Collegeville, Pennsylvania; Pfizer Vaccines, Dublin, Dublin, Ireland

## Abstract

**Background:**

Beyond the burden of acute respiratory infection, RSV can cause cardiorespiratory disease exacerbations and trigger cardiovascular events in adults. Information on these associations is limited, particularly following the acute illness phase. We evaluated the risk of common cardiorespiratory events during and following RSV hospitalization in adults.

**Figure:**
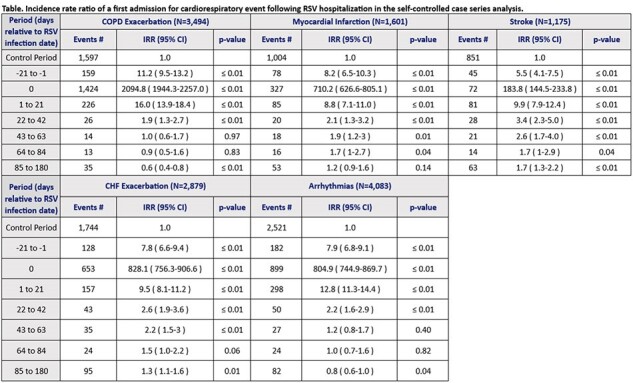

**Methods:**

We conducted a self-controlled case series analysis using data from administrative claims and electronic health records from Optum’s de-identified Market Clarity database. Adults (≥18 years) with 6 months of baseline continuous enrollment and an RSV-related hospitalization (RSV diagnosis or RSV+ test in the inpatient setting) from 01Jan17–30Jun23 (observation period) were included. Patients with the first admission for chronic obstructive pulmonary disease exacerbations (COPD-E), myocardial infarction (MI), stroke, congestive heart failure exacerbations (CHF-E), and arrhythmias during this period were identified using diagnosis codes. The risk period was defined as 0–180 days after RSV infection, including subintervals of risk periods and pre-risk periods. The remaining time outside of the 180-day risk period and the 21-day pre-risk period during the observation period formed the control period. Conditional Poisson regression adjusting for time-varying age (5-year intervals) was performed to determine the incidence rate ratio (IRR) for each event following RSV infection.

**Results:**

Among 8,181 adults hospitalized with RSV, 3,494 had COPD-E 1,601 MI, 1,175 stoke, 2,879 CHF-E, and 4,083 arrhythmias during the entire observation period. The IRR during days 1–21 compared to the control period following RSV infection was 16.0 (95% CI 13.9, 18.4), 8.8 (95% CI 7.1,11.0), 9.9 (95% CI 7.9, 12.4), 9.5 (95% CI 8.1, 11.2), and 12.8 (95% CI 11.3, 14.4), respectively, for COPD-E, MI, stroke, CHF-E, and arrhythmias. IRRs were elevated for all outcomes for 42 days and attenuated over time but remained elevated for up to 180 days for stroke and CHF-E.

**Conclusion:**

There is an increased risk of acute cardiorespiratory events and exacerbations following RSV infection. These cardiorespiratory events should be considered as part of the burden of RSV infection when evaluating RSV preventive interventions.

**Disclosures:**

**Caihua Liang, MD, PhD**, Pfizer: Stocks/Bonds (Private Company) **Jennifer Judy, MS, PhD**, Pfizer: Employee|Pfizer: Stocks/Bonds (Public Company) **Negar Aliabadi, MD, MS**, Pfizer Inc: employment|Pfizer Inc: Stocks/Bonds (Public Company) **Reiko Sato, PhD**, Pfizer Inc: employee|Pfizer Inc: Stocks/Bonds (Private Company) **Erica Chilson, PharmD**, Pfizer Inc: Employee|Pfizer Inc: Stocks/Bonds (Public Company) **Xin Zhao, MS**, Pfizer: Xin Zhao is an employee of Genesis Research Group which received compensation from Pfizer for conducting this study. **Bradford D. Gessner, M.D., M.P.H.**, Pfizer: Employee|Pfizer: Stocks/Bonds (Public Company) **Elizabeth Begier, MD, M.P.H.**, Pfizer Vaccines: Employee|Pfizer Vaccines: Stocks/Bonds (Private Company)

